# Utility of CT Perfusion Imaging in Patients With Vertebral Artery Stenosis Treated With Balloon Expandable Stent

**DOI:** 10.3389/fneur.2021.650887

**Published:** 2021-03-17

**Authors:** Wei Wei, Chong Song, Xuqin Li, Dianshi Jin

**Affiliations:** Department of Neurosurgery, Affiliated Dalian Municipal Central Hospital, Dalian Medical University, Dalian, China

**Keywords:** vertebral artery stenosis, stent, CT perfusion, cerebral blood flow, mean transmit time, cerebral blood volume

## Abstract

**Objective:** To investigate the clinical value of CT perfusion (CTP) imaging in vertebral artery stenosis stenting, so as to provide the basis for preoperative and postoperative evaluation. Ninety-seven patients with vertebral artery stenosis were accepted for endovascular stenting between Jan 2016 and Jan 2020. CT angiography, Digital Subtraction Angiography, and CTP were performed pre-operation and post-operation. The cerebral blood volume (CBV), cerebral blood flow (CBF), and mean transmit time (MTT) between the health and affected sides were analyzed statistically, and the imaging results pre- and post-operation were evaluated. The stenosis was relieved by endovascular stents in all 97 patients without serious complications. The abnormal perfusion was observed in 66 patients (68%). The differences in CBF and MTT between the diseased side and healthy side were statistically significant (*P* < 0.05). Compared with the preoperative imaging, the postoperative CTP was improved in 59 patients (89%). The differences in CBF and MTT between pre-operation and post-operation were statistically significant (*P* < 0.05). But there was no significant difference in CBV. CTP can sensitively reflect the perfusion of brain, and can also be used for preoperative and postoperative evaluation of vertebral artery stenting. It may be helpful as an adequate indicator of vertebral artery stenosis stent surgery.

## Introduction

Stroke is a serious threat to human health, ~87% of all strokes are ischemic strokes (1), among which posterior circulation (PC) ischemic stroke accounts for about 20%. Nearly 25% of patients with vertebral artery stenosis or basilar artery stenosis have progressed cerebral infarction, and symptomatic vertebrobasilar artery stenosis is especially prone to recurrent stroke (2). Vertebral artery (VA) stenosis can be effective treated with balloon dilatation or vertebral artery stenting (3, 4). Very low complication rates (1–1.5%) have been suggested with stenting for extracranial VA stenosis, however higher complication rates for intracranial stenosis about 7–10% (5–7). Patients with vertebral artery stenosis may only show dizziness, but dizziness can be caused by several diseases. This symptom is not very objective. Even if vertebral artery stenosis causes cerebral ischemia, CT and MRI do not show ischemic lesions due to the compensation of collateral circulation. So far, the indication of vertebral artery stenosis stent surgery has often been determined by clinical symptoms which sometimes may be not obvious and easily neglected, the degree of imaging stenosis, and lack of objective indicators. CT perfusion (CTP) imaging could quantitatively analyze the cerebral blood flow through the cerebral blood volume (CBV), the cerebral blood flow (CBF), and mean transit time (MTT), which were used to show the cerebral blood supply and perfusion of ischemic cerebrovascular disease (8, 9). Therefore, CTP may be helpful as an adequate indicator of vertebral artery stenosis stent surgery.

## Methods

### Patient Data

From Jan 2016 to Jan 2020, 97 patients in Dalian Municipal Central Hospital Affiliated of Dalian Medical University were diagnosed with vertebral artery stenosis by CT angiography (CTA) or Digital Subtraction Angiography (DSA), and received endovascular stent treatment in our department.

### Inclusion and Exclusion Criteria

Inclusion criteria: (1) The unilateral vertebral artery opening stenosis which was at least 50% was confirmed by CTA or DSA, with posterior circulation ischemia symptoms. (2) The vertebral artery stenosis is on the dominant side. (3) No other intracranial vascular diseases. Exclusion criteria: (1) The unilateral vertebral artery stenosis was <50%. (2) Non-dominant side vertebral artery stenosis was >50%. (3) Vertebral artery intracranial segment with multiple stenosis.

### Perioperative Management

In our study DSA or CTA and CT perfusion (CTP) were arranged within 3 days before and after surgery to evaluate the ischemic degree of the vertebral artery. All patients were injected with 40 ml iodixanol with a high-pressure syringe in the median cubital vein, scanned CT with 5 mm, and reconstructed with 1.5 mm slice thickness. The reconstruction and perfusion analysis of images were carried out using the advantage work station version 4.5 software (AW45, USA). The CBF, CBV, and MTT of the diseased side and the healthy side, as well as pre-operation and post-operation were measured. DSA procedure: After successful local anesthesia, Seldinger technique was used to puncture the radial artery or femoral artery, and a 6F artery sheath was routinely used to perform bilateral subclavian artery and common carotid artery angiography, respectively. The angiography tube could be placed in the subclavian artery to better display the vertebral artery opening, and the degree and length of vascular stenosis were shown at an appropriate Angle. According to North American symptomatic carotid endarterectomy trial (NASCET), the vertebral artery stenosis was graded, the moderate stenosis rate was 50–70%, and the severe stenosis rate was 70–99%. All patients started receiving aspirin (100 mg/d) and clopidogrel (75 mg/d) daily at least 3 days before stenting or a loading dose of aspirin and clopidogrel of 300 mg, respectively, if emergency surgery was needed. After surgery, they were maintained on aspirin (100 mg/d) and clopidogrel (75 mg/d) for no <6 months.

### Vertebral Artery Stenting Procedure

All the patients were confirmed by DSA or CTA as vertebral artery origin stenosis. The patients were treated with Apolo balloon expandable stent (Shanghai minimally invasive company) or Vertebral artery balloon expandable stent (Boston Scientific, USA).

After anesthesia, with the Seldinger technique, a right femoral artery puncture was performed to place an 8F arterial sheath, the patients received intravenous injection of 3,000 U low-molecular-weight heparin sodium for systemic heparinization. Then a Gateway balloon was chosen and placed across the stenotic segment for balloon dilation. After successful expansion, the balloon was withdrawn. According to the normal diameter and stenosis length of the vertebral artery, the size of the stent was selected. Then the stent was placed in the lesioned vessel. When radiographic examination reported the degree of stenosis improved significantly, the stent delivery system was removed. Intracranial angiography was repeated after observation for 30 min, if no abnormalities were found, the operation was finally finished.

### Statistical Methods

All analyses were performed using the SPSS 25.0 software (SPSS Inc., Chicago, IL, USA). Measurement data is expressed in $\bar{\text{x}}$ ± s. The parameters of the diseased side and the healthy side, as well as pre-operation and post-operation, were compared by *t*-test.

## Results

A total of 97 patients with vertebral artery stenosis which accepted vertebral artery stenting were enrolled in this study. There were 45 males and 42 females, aged from 50 to 80 years old (median age 65 years). Clinical manifestations: dizziness in 60 cases, walking instability in 22 cases, headache with nausea in 15 cases. Preoperative DSA confirmed moderate stenosis in 47 cases, severe stenosis 50 cases. All patients were examined by CTP in peri-operation period, the abnormal perfusion was observed in 66 patients (68%). The positive rate of abnormal perfusion area corresponding to clinical symptoms found in MTT was 100%. And the abnormal perfusion area was found in 40 cases on CBF, the positive rate was 61%. In CBV the positive rate was only 26%. And total 58 cases (88%) had significant improvement in CT perfusion after vertebral artery stenting. Compared with the healthy side, the difference of CBF and MTT in the diseased side was statistically significant. Besides, compared with pre-operation, CBF was significantly increased and MTT was decreased after vertebral artery stent implantation. But there was no significant difference in CBV (The detailed discrepancy was shown in Tables 1, 2).

**Table 1:** Comparison of CTP perfusion parameters between diseased side and healthy side.

**Parameter**	**Healthy side**	**Diseased side**	***t*-value**	***P-*value**
CBF [ml/(100 g·min)], ($\bar{x}\pm$ s)	45.47 ± 6.69	35.79 ± 5.49	−8.869	0.000^*^
CBV (ml/100 g), ($\bar{x}$ ± s)	1.82 ± 0.25	1.92 ± 0.38	1.63	0.106
MTT (s)	3.86 ± 0.26	4.78 ± 0.56	11.79	0.000^*^

* *P < 0.05, with statistical significance*.

**Table 2:** Comparison of CTP perfusion parameters between pre-operation and post-operation.

**Parameter**	**Pre-operation**	**Post-operation**	***t*-value**	***P*-value**
CBF [ml/(100 g·min)], ($\bar{x}$ ± s)	35.16 ± 4.09	46.79 ± 5.55	−13.70	0.000^*^
CBV (ml/100 g), ($\bar{x}$ ± s)	1.71 ± 0.31	1.78 ± 0.21	−1.61	0.100
MTT (s)	4.43 ± 0.56	3.96 ± 0.21	6.34	0.000^*^

* *P < 0.05, with statistical significance*.

## Discussion

In clinic, vertebrobasilar artery stenosis was a common disease leading to recurrent PC strokes, the main treatment for this disease was stent implantation. At present, the indications of stenting for vertebral artery stenosis are usually determined by whether the degree of stenosis is more than 50% which is detected by CTA or DSA examination before operation, and corresponding clinical symptoms such as dizziness, walking unsteadily, or the other symptoms (10–13). However, there was no objective assessment of the blood flow in the posterior circulation. The CTP examination often used MTT, CBF, and CBV to evaluate the perfusion state of brain tissue. Sometimes, there may be no abnormal expression of MRI and CT before clinical onset of cerebral infarction. Several studies suggested that CTP, MTT, and CBF can quantitatively and effectively evaluate cerebral hemodynamic abnormalities before cerebral infarction (14). The changes of the above parameters represented corresponding brain function, and CTP was very sensitive to early cerebral hemodynamic changes (15–18). In our study, the abnormal perfusion was observed in 66 patients (68%). Among them MTT was 100%, which was consistent with the corresponding clinical symptoms. And the abnormal perfusion area was found in 40 cases on CBF (61%) and 17 cases on CBV (26%). It was shown that CTP could sensitively demonstrate the abnormal perfusion of posterior circulation, especially MTT. Among the above three parameters, MTT was the most sensitive in early detection of perfusion abnormalities. The results of our study were similar to those of previous studies, which suggested that CTP may be utilized in the posterior circulation (19–22), and MTT parameters are more sensitive than CBF or CBV for identifying lacunar infarcts (23). In addition, in our study 31 patients with the stenosis at the opening of the vertebral artery more than 50% confirmed by CTA or DSA, but the changes of CBF and CBV were not obvious. It was speculated that the mechanism may be as follows: There are many collateral circulations which were normally not open in cerebral vessels. However, when chronic stenosis or occlusion occurs, the collateral vessels proliferate, the distal vessels dilate, and the blood flow increases to maintain the normal level of CBF. In addition, even though the feeding artery was completely occluded, the collateral circulation has already been established in the process of chronic occlusion, which could also maintain normal cerebral blood flow and cerebral perfusion. Therefore, patients did not necessarily benefit from stenting in such patients. Besides, when the vertebral artery stenosis occurred in the non-dominant blood supply side, the cerebral blood flow and perfusion was not affected, and there was also no indication of vertebral artery stenting.

Based on our findings, DSA or CTA may be more sensitive as an indicator of vertebral artery stenosis stent surgery compared with CT imaging, and as we know that DSA was the gold standard for the diagnosis of cerebrovascular diseases, this could accurately demonstrate the degree of cerebral vascular stenosis and the state of cerebral vascular collateral circulation. However, it was an invasive examination and expensive, so sometimes it was not easy for patients to accept. Although CTA was a non-invasive examination, its imaging was sometimes affected by bone calcification, which is not as accurate as DSA. Compared with the above two examinations, CTP could directly reflect the cerebral blood flow perfusion, rather than simply taking the degree of vascular stenosis as the surgical indication (24–26). In our study, there were a total of 66 patients with abnormal preoperative CT perfusion, and 58 of them has been improved significantly after operation. This suggested that CTP could not only evaluate the effect of vertebral artery stenting, but also observe the improvement of cerebral perfusion. In summary, CTP can sensitively reflect the perfusion of the brain, and can also be used for preoperative and postoperative evaluation of vertebral artery stenting. So, it may be helpful as an adequate indicator of vertebral artery stenosis stent surgery.

## Data Availability Statement

The raw data supporting the conclusions of this article will be made available by the authors, without undue reservation.

## Ethics Statement

The studies involving human participants were reviewed and approved by Dalian Municipal Central Hospital. The patients/participants provided their written informed consent to participate in this study.

## Author Contributions

WW and CS collected the data and wrote the original manuscript draft. WW analyzed the data. XL and DJ revised the manuscript and approved the final version. All authors contributed to the article and approved the submitted version.

## Conflict of Interest

The authors declare that the research was conducted in the absence of any commercial or financial relationships that could be construed as a potential conflict of interest.
